# Geographic, Demographic, and Socioeconomic Disparities and Factors Associated With Cancer Literacy in China: National Cross-sectional Study

**DOI:** 10.2196/43541

**Published:** 2023-02-17

**Authors:** Siyi He, He Li, Maomao Cao, Dianqin Sun, Fan Yang, Xinxin Yan, Shaoli Zhang, Changfa Xia, Yiwen Yu, Liang Zhao, Jufang Shi, Ni Li, Xue Qin Yu, Wanqing Chen, Jie He

**Affiliations:** 1 Office of Cancer Screening National Cancer Center/National Clinical Research Center for Cancer/Cancer Hospital, Chinese Academy of Medical Sciences and Peking Union Medical College Beijing China; 2 The Daffodil Centre - a joint venture with Cancer Council NSW The University of Sydney Sydney Australia; 3 Thoracic Surgery Department National Cancer Center/National Clinical Research Center for Cancer/Cancer Hospital, Chinese Academy of Medical Sciences and Peking Union Medical College Beijing China

**Keywords:** health literacy, cancer literacy, cross-sectional study, Healthy China Initiative, cancer control

## Abstract

**Background:**

Cancer literacy is associated with several health-related behaviors and outcomes. However, there is still a lack of nationwide surveys for cancer literacy in China.

**Objective:**

This study aims to evaluate cancer literacy in China, explore disparities, and provide scientific evidence for policy makers.

**Methods:**

A cross-sectional survey was conducted in mainland China in 2021 using the multistage probability proportional to the size sampling method. Both the reliability and validity of the questionnaire were evaluated. The awareness levels were adjusted by sampling weights and nonrepresentativeness weights to match the actual population distributions. The Rao-Scott adjusted chi-square test was applied to test geographic, demographic, and socioeconomic disparities. A generalized linear model was used to explore potential factors.

**Results:**

A total of 80,281 participants aged 15-74 years were finally enrolled from 21 provinces, with an overall response rate of 89.32%. The national rate of cancer literacy was 70.05% (95% CI 69.52%-70.58%). The rates were highest regarding knowledge of cancer management (74.96%, 95% CI 74.36%-75.56%) but were lowest regarding basic knowledge of cancer (66.77%, 95% CI 66.22%-67.33%). Cancer literacy was highest in East China (72.65%, 95% CI 71.82%-73.49%), Central China (71.73%, 95% CI 70.65%-72.81%), and North China (70.73%, 95% CI 68.68%-72.78%), followed by Northeast (65.38%, 95% CI 64.54%-66.22%) and South China (63.21%, 95% CI 61.84%-64.58%), whereas Southwest (59.00%, 95% CI 58.11%-59.89%) and Northwest China (57.09%, 95% CI 55.79%-58.38%) showed a need for improvement. Demographic and socioeconomic disparities were also observed. Urban dwellers, the Han ethnic group, and population with higher education level or household income were associated with prior knowledge. The questionnaire showed generally good internal and external reliability and validity.

**Conclusions:**

It remains important for China to regularly monitor levels of cancer literacy, narrow disparities, and strengthen health education for dimensions with poor performance and for individuals with limited knowledge to move closer to the goal of Healthy China 2030.

## Introduction

Cancer is a global issue that threatens public health, and 23.7% of new cancer cases and 30.2% deaths occurred in China in 2020 [1]. Meanwhile, regional and population disparities are evident in terms of the burden of cancer across China [2]. Cancer ranked the first and the third cause of death in urban and rural China in 2020, respectively, and more cancer-related resources, including qualified medical workers, high-level medical institutions, and health input, were allocated in East China [3]. Faced with this situation, the Chinese government has issued a series of documents [4-6] aiming to gradually promote comprehensive strategies to slow down the growth of cancer. Constant efforts have also been made to encourage health equity by increasing funds and enacting policies.

**Figure 1:**
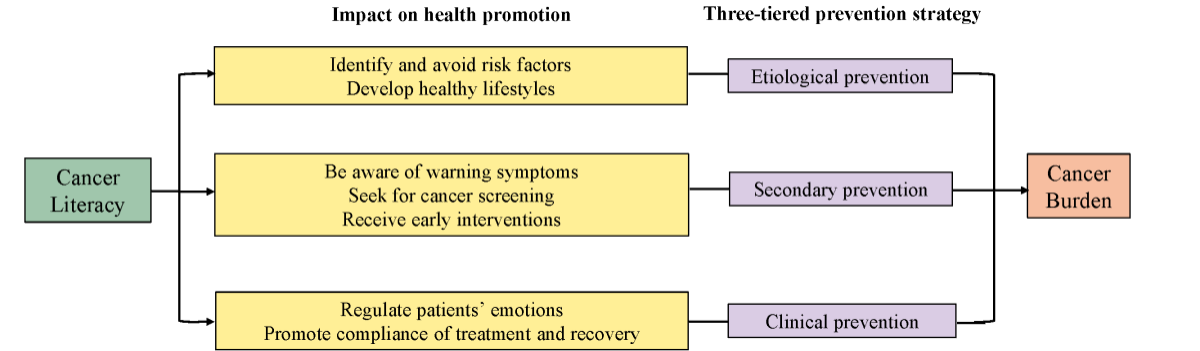
Cancer literacy might be associated with the burden of cancer through a 3-tiered prevention strategy.

Policy makers in the United States have recognized the significance of health literacy, which may be a potential intervenable factor to reduce health disparities with decreased financial burdens on the health system [7,8]. Cancer literacy refers to all the knowledge a layperson needs to possess to understand the information and advice the health system has to offer with regard to preventing, diagnosing, and treating cancer [9]. Up to now, several studies have reported a consistent association between health literacy and some health-related behaviors or outcomes, including physical exercise, cancer screening, and mortality [8,10-12]. Therefore, the Chinese government has called for improvement in cancer literacy, with the aim of finally decreasing the burden of cancer on the basis of a 3-tiered prevention strategy (Figure 1) [13,14].

Specifically, the Healthy China Initiative (2019-2030), the guiding principle for national health undertakings in China in the next decade, proposed phased targets for cancer literacy of 70% and 80% by 2022 and 2030, respectively [5]. The National Health Commission of China has formulated a series of the Three-year Action Plan for Cancer Prevention and Control, which repeatedly emphasized urgency and significance of improving cancer literacy [4,13]. As 1 of the 2 indicators for the assessment of government work in cancer control action in the Healthy China Initiative, the level of cancer literacy among the Chinese population needs to be evaluated to indicate the current status of progress in 2022 and to further promote the accomplishment of the next goal. However, there is still a lack of reliable measurement tools in China, and thus, national data on cancer literacy are not available. To fill this gap, the primary aim of this study was to evaluate the current level of cancer literacy in China using a well-developed questionnaire. The second aim was to assess disparities among regions and populations to provide references for health education.

## Methods

### Ethics Approval

This study protocol was approved by the ethics committee of the cancer hospital of the Chinese Academy of Medical Sciences (NCC-007739). We followed the Strengthening the Reporting of Observational Studies in Epidemiology reporting guideline [15].

### Participants and Sample Size

A national cross-sectional survey was administered in 7 administrative divisions of mainland China using the multistage probability proportional to size sampling (PPS) method in 2021. Residents aged 15-74 years were selected, except for those registered in a collective residence. At least 2152 samples had to be included in each province to ensure their evaluations of cancer literacy (see details in Multimedia Appendix 1).

### Sampling Method

The sampling process was divided into 6 stages in this national survey (Multimedia Appendix 1). Briefly, the primary sampling units of this study were provinces, and a total of 21 provinces were selected from 7 administrative units using the PPS method. The secondary sampling units (SSUs) were county-level administrative units, and the whole survey was conducted in urban and rural areas, which were classified according to the document issued by the Ministry of Civil Affairs. The PPS method was applied based on the *2020 China Statistical Yearbook* [14], and finally 3 streets or towns and 6 neighborhood or village committees were randomly selected in each SSU. Simple random sampling was used to select households based on the electronic geographic listings of community households. One eligible family member was identified with the Kish grid method and selected to complete a questionnaire survey [16]. Participants were reached and reviewed by attempts tried per household.

### Survey Tool

The tool applied in this study was designed by the National Cancer Center of China and contains a 3-level index framework constructed based on an extensive literature, public document, and expert opinions (Multimedia Appendix 2). The questionnaire consists of 13 true-or-false items, 13 single-choice items, and 11 multiple-choice items, and it covers 5 main dimensions: basic knowledge (9 scores, 24.3%), primary prevention (9 scores, 24.3%), early detection and treatment (10 scores, 27.0%), treatment (6 scores, 16.3%), and recovery of cancer (3 scores, 8.1%). Each item corresponds to the information recommended by the Chinese Health Commission after several rounds of deliberations, and all the items combined form a comprehensive assessment of individuals’ cognition, attitude, skills, and behavior choices for cancer prevention and treatment [17]. In addition, basic information was also investigated, mainly age, sex, education level, ethnicity, family history of cancer, smoking status, annual income, and marital status.

### Data Weighting and Calculation

For all items, 1 score was given for a complete correct answer, zero score was given for a wrong or partially correct answer, and the final score was defined as the sum of all the scores. Cancer literacy at the population level was calculated as the sum of the final scores of all respondents divided by the number of items to be answered. Meanwhile, the cancer literacy estimates were adjusted by the product of design weights and nonrepresentativeness weights, considering the inevitable inconsistencies of population distribution between the survey sample and the actual population (see details in Multimedia Appendix 1). SE values and the corresponding 95% CIs were also calculated.

### Statistical Analysis

The completed questionnaires were included in the final analysis. Continuous variables are presented as the mean (SD) or median (IQR), and categorical variables are reported as counts and percentages. The internal and external reliability and validity of the questionnaire were evaluated. Item response theory (IRT) 2-parameter logistic analysis was used to evaluate the discrimination (*a* parameter) and difficulty (*b* parameter) of each item by dimension. The values of the Human Development Index (HDI), age-standardized incidence rates, and mortality rates for 7 administrative divisions were extracted to explore potential associations with cancer literacy [2,18]. The response rates were estimated based on both the unit nonresponse and the item nonresponse. R (version 4.1.0; R Foundation for Statistical Computing) and SAS (version 9.4; SAS Institute Inc) software were used to conduct data analysis. The Rao-Scott adjusted chi-square test was used to compare cancer literacy among subgroups, which is a modified chi-square test method for survey data by improving the performance in the goodness-of-fit problem and avoiding misleading results generated by clustering in the survey design [19,20]. When any assumptions of linear regression were violated (Linear relationship, Independence, Homoscedasticity, and Normality) [21], the levels of cancer literacy were grouped according to quartiles, and the multivariable logistic regression analysis was applied to explore the potential factors of cancer literacy, with adjustments of all the background variables except self-reported health status. A 2-sided *P*<.05 was considered statistically significant.

## Results

### Basic Characteristics

By December 31, 2021, a total of 80,281 questionnaires had been completed in mainland China, with an overall response rate of 89.32% (Table S1, Multimedia Appendix 3). The median age of all the participants was 46 (IQR 34-56) years, and the population with registered permanent residences in urban areas accounted for 51.24%, which was representative of Chinese citizens according to the Sixth Census in China. As shown in Table S2 (Multimedia Appendix 3), the survey sample overrepresented females (57.49%) and those with high levels of education (associate degree and above, 39.17%), whereas Western China (Southwest and Northwest, 14.58%) was underrepresented compared with the respective values of 21.19% from census data. Multistep adjustments of the above variables were carried out to ensure the representativeness of the main findings. Additionally, most participants were married (81.11%), never smokers (74.75%), and had no family history of cancer (68.50%). There were no logical errors in the collected questionnaires during random sampling checks, indicating good quality control of this survey.

### Assessments of the Questionnaire

A panel composed of 26 experts in clinical medicine, preventive medicine, health economics, imaging diagnostics, and immunology assessed the difficulty and significance of the questionnaire framework, and the tool generally showed satisfactory content validity (Tables S3-S5, Multimedia Appendix 4). The cancer literacy rate of medical practitioners was different from that of nonmedical practitioners, that is, 80.15% (95% CI 76.08%-84.21%) and 69.83% (95% CI 69.29%-70.37%), respectively, suggesting reasonable external validity. The assessment of the internal consistency yielded an overall Cronbach coefficient of .92. The coefficient values of the 5 dimensions were 0.69, 0.79, 0.77, 0.76, and 0.59, respectively, and the former 4 dimensions were internally consistent on their own. Retest questionnaires were obtained 2 weeks later, and the correlation between the 2 rounds of testing was 0.75, indicating acceptable test-retest reliability. In addition, Table S6 (Multimedia Appendix 4) reports the IRT parameter estimates. Discrimination parameters for all the items ranged between 0.37 and 3.67, and the difficulty estimates were also in the reasonable range [22].

### Overall Levels of Cancer Literacy

The national level of cancer literacy in China was 70.05% (95% CI 69.52%-70.58%). The awareness rates in terms of treatment and recovery of cancer were relatively high at 74.96% (95% CI 74.36%-75.56%) and 71.00% (95% CI 70.25%-71.76%), respectively, followed by the level of knowledge of primary prevention (70.81%, 95% CI 70.16%-71.46%). However, the corresponding levels of basic knowledge and early detection for cancer were below the target at 66.77% (95% CI 66.22%-67.33%) and 69.16% (95% CI 68.61%-69.71%), respectively. Detailed rates of cancer literacy in terms of more specific dimensions are shown in Table S7 (Multimedia Appendix 5). Levels of cancer literacy in terms of the early diagnosis of cancer, positive beliefs in cancer, and the physiological recovery were relatively low at 59.05% (95% CI 58.32%-59.79%), 63.35% (95% CI 62.72%-63.99%), and 66.13% (95% CI 65.16%-67.10%), respectively.

### Disparities in Cancer Literacy Levels

**Figure 2:**
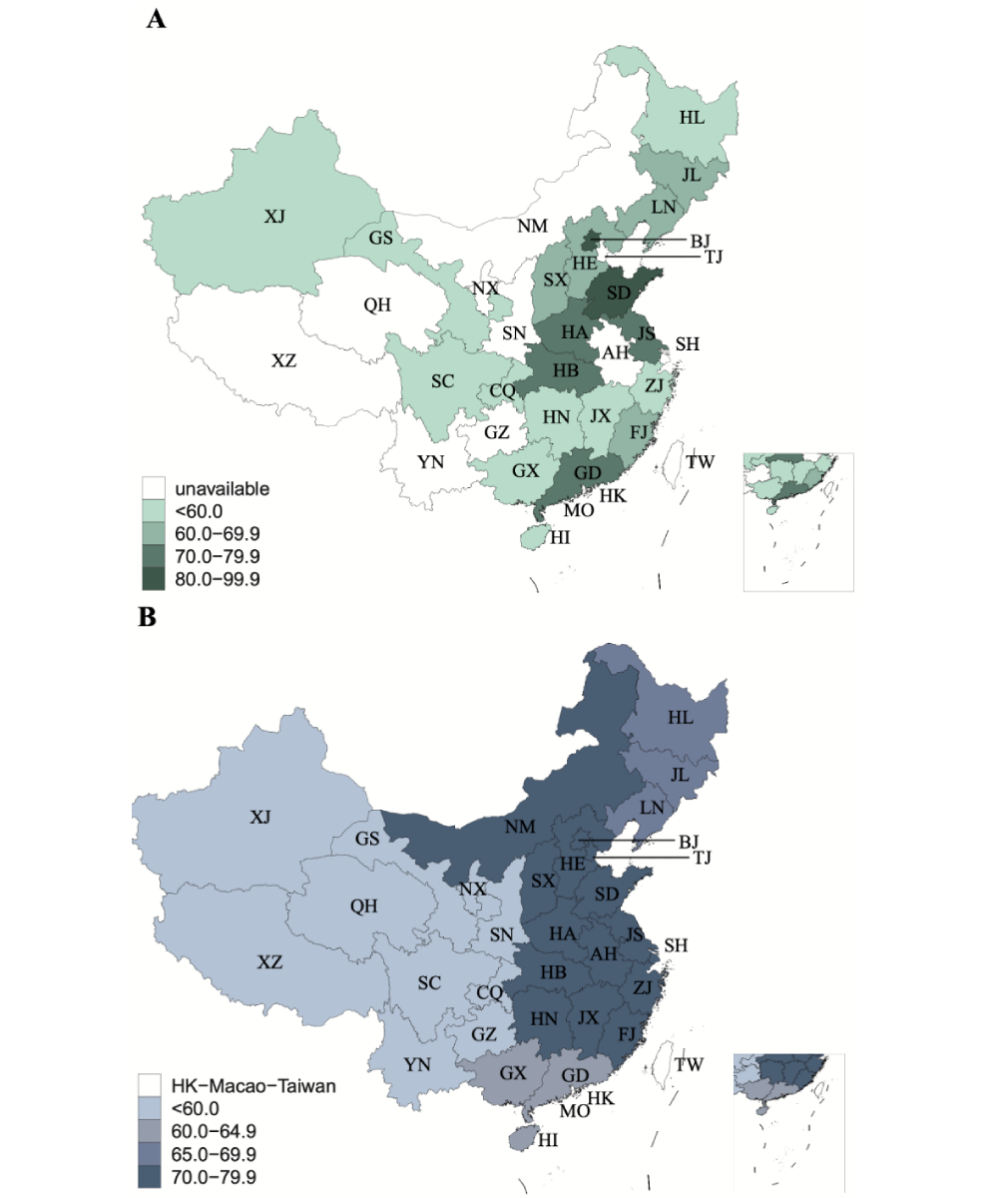
Geographic disparities among provinces (A) and among administrative divisions (B) in terms of the rates of cancer literacy in China, 2021. The provinces included in each administrative division were determined following the government document of China, and all the provinces were allocated to 7 administrative divisions. AH, Anhui; BJ, Beijing; CQ, Chongqing; FJ, Fujian; GD, Guangdong; GS, Gansu; GX, Guangxi; GZ, Guizhou; HA, Henan; HB, Hubei; HE, Hebei; HI, Hainan; HK, Hong Kong; HL, Heilongjiang; HN, Hunan; JL, Jilin; JS, Jiangsu; JX, Jiangxi; LN, Liaoning; MO, Macao; NM, Inner Mongolia; NX, Ningxia; QH, Qinghai; SC, Sichuan; SD, Shandong; SH, Shanghai; SN, Shaanxi; SX, Shanxi; TJ, Tianjin; TW, Taiwan; XJ, Xinjiang; XZ, Tibet; YN, Yunnan; ZJ, Zhejiang.

There were distinct discrepancies among the geographical distributions of levels of cancer literacy (Figure 2). In general, the rates were highest in the population residing in East China (72.65%, 95% CI 71.82%-73.49%), Central China (71.73%, 95% CI 70.65%-72.81%), and North China (70.73%, 95% CI 68.68%-72.78%), followed by Northeast (65.38%, 95% CI 64.54%-66.22%) and South China (63.21%, 95% CI 61.84%-64.58%), and lowest in Southwest (59.00%, 95% CI 58.11%-59.89%) and Northwest China (57.09%, 95% CI 55.79%-58.38%). In general, the disease burden of cancer decreased with improvements in cancer literacy, and superior cancer literacy seems to be associated with a higher HDI value (Table S8, Multimedia Appendix 5).

**Figure 3:**
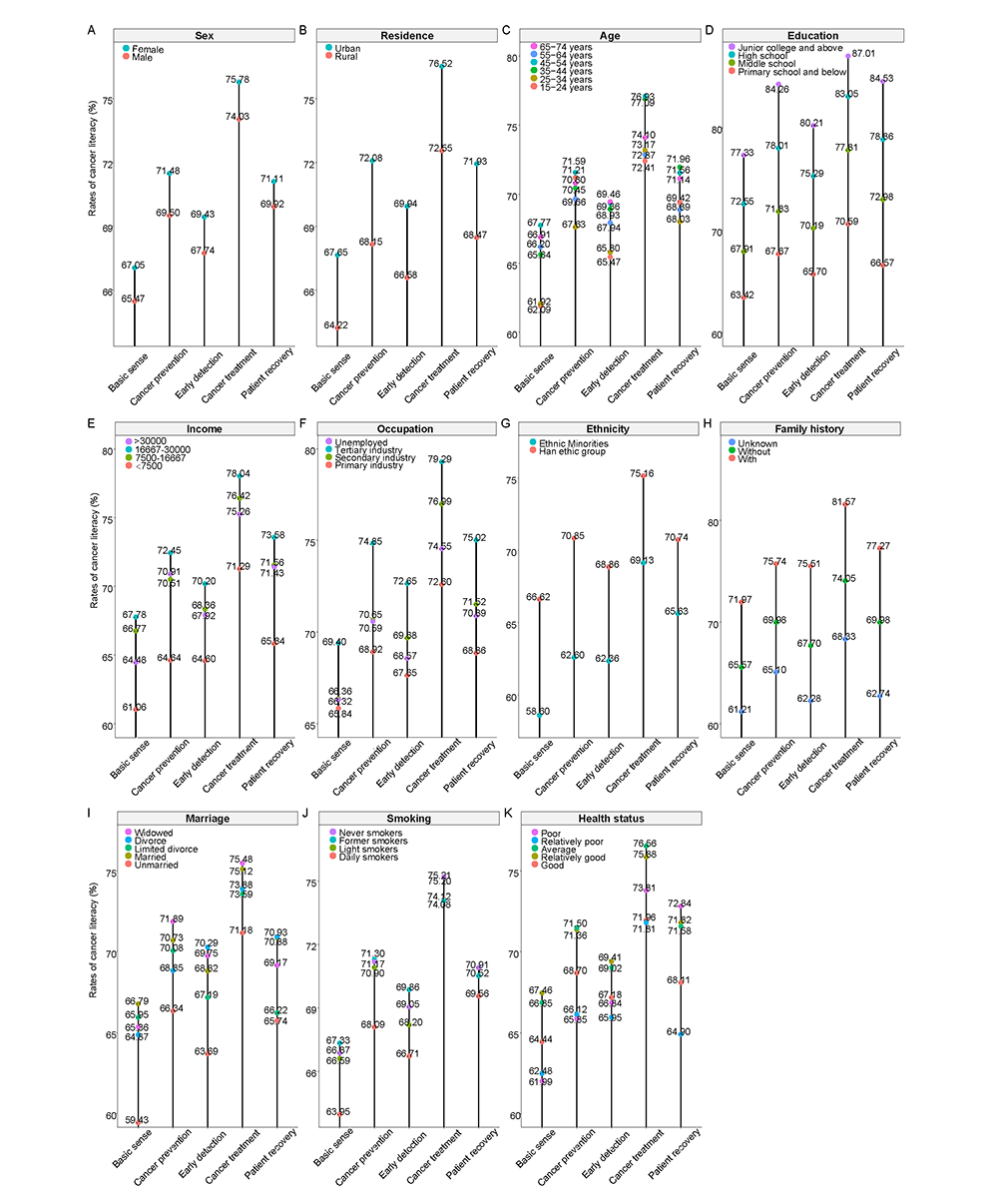
Demographic and socioeconomic disparities in terms of the overall and dimension-specific rates of cancer literacy in China, 2021.

Demographic and socioeconomic disparities in population distributions were also observed for cancer literacy within China (Table 1, Figure 3). Briefly, urban dwellers (vs rural dwellers, 71.25% vs 68.45%) and females (vs males, 70.73% vs 69.45%) had prior knowledge of cancer prevention and control. The rates also increased as the education level (*P*<.001) and annual household income (*P*<.001) increased. Although there was no significant trend in the rates of cancer literacy among age groups, individuals aged 35-54 years showed the highest overall and dimension-specific rates, and elderly individuals had better knowledge in terms of basic knowledge and early detection and treatment of cancer than those younger than 35 years. In addition, the levels of cancer literacy remained higher among people of the Han ethnic group (vs ethnic minorities, 70.43% vs 63.42%), married individuals (vs unmarried individuals, 70.38% vs 65.38%), never smokers (vs daily smokers, 70.58% vs 67.96%), and individuals with a family history of cancer (vs individuals without or with an unknown family history, 75.93% vs 69.45% or 64.34%). After multivariable adjustment, the above variables remained significantly associated with cancer literacy (Table 2).

**Table 1:** The levels of cancer literacy by participant characteristics.

Characteristics		Sample size (n)	Cancer literacy, % (range)	*P* value	
**Sex**					.02
	Male	34,127	69.45 (68.72-70.19)		
	Female	46,154	70.73 (69.96-71.51)		
**Education level**					<.001
	Primary school and below	12,778	66.80 (65.79-67.82)		
	Middle school	19,827	72.05 (71.56-72.54)		
	High school	16,231	77.45 (77.02-77.87)		
	Associate degree and above	31,445	82.25 (81.94-82.55)		
**Type of registered permanent residence**					<.001
	Rural	41,138	68.45 (68.05-68.85)		
	Urban	39,143	71.25 (70.36-72.14)		
**Age (years)**					<.001
	15-24	5292	68.03 (64.52-71.54)		
	25-34	15,183	67.77 (65.35-70.19)		
	35-44	17,915	71.01 (69.64-72.38)		
	45-54	19,179	71.41 (70.48-72.33)		
	55-64	14,146	68.93 (67.94-69.91)		
	65-74	8566	70.19 (68.95-71.42)		
**Ethnicity**					<.001
	Han ethnic group	74,375	70.43 (69.88-70.98)		
	Ethnic minority	5856	63.42 (60.90-65.94)		
**Marital status**					.20
	Unmarried	10,855	65.38 (62.27-68.48)		
	Married	65,112	70.38 (69.83-70.94)		
	Separated	333	68.86 (63.28-74.43)		
	Divorced	1954	69.12 (66.16-72.08)		
	Widowed	1673	70.85 (68.40-73.30)		
**Occupation**					<.001
	Primary industry	24,471	68.37 (67.65-69.09)		
	Secondary industry	4919	70.47 (68.42-72.52)		
	Tertiary industry	35,220	73.68 (72.43-74.92)		
	Unemployed	14,828	69.65 (68.56-72.52)		
**Smoking status**					<.001
	Daily smoker	12,187	67.96 (66.86-69.05)		
	Light smoker	2563	70.97 (67.87-74.08)		
	Former smoker	5282	71.42 (69.51-73.33)		
	Never smoker	60,012	70.58 (69.95-71.21)		
**Self-reported health status**					.001
	Good	24,216	68.37 (67.32-69.42)		
	Relatively good	29,131	70.84 (69.93-71.75)		
	Average	23,736	71.11 (70.22-72.00)		
	Relatively poor	2631	67.08 (64.28-69.87)		
	Poor	549	67.55 (61.89-73.21)		
**Family history of cancer**					<.001
	Yes	18,129	75.93 (74.90-76.96)		
	No	54,989	69.45 (68.84-70.05)		
	Unknown	6801	64.34 (62.27-66.41)		
**Annual household income per capita**					<.001
	<7500 CNY (<US $1117.00)	15,198	65.41 (63.92-66.89)		
	7500-16,667 CNY (US $1117.00-2482.28)	21,313	70.51 (69.56-71.46)		
	16,667-30,000 CNY (US $2482.28-4468.01)	12,817	71.88 (70.79-72.97)		
	>30,000 CNY (>US $4468.01)	17,323	70.12 (68.75-71.49)		

**Table 2:** Multivariate analysis of the associations between the included variables and the levels of cancer literacy.

Variables		OR^a^ Level 2^b^	*P* value	OR^a^ Level 3^b^	*P* value	OR^a^ Level 4^b^	*P* value
**Sex**							
	Male	1 (Reference)					
	Female	1.06	.18	1.07	.12	1.20	<.001
**Age (years)**							
	15-24	1 (Reference)					
	25-34	1.12	.39	0.84	.19	0.79	.08
	35-44	1.20	.14	0.89	.32	1.13	.35
	45-54	1.27	.048	0.95	.65	1.13	.33
	55-64	1.106	.62	0.76	.02	0.95	.70
	65-74	1.00	.97	0.88	.27	1.25	.07
**Smoking status**							
	Daily smoker	1 (Reference)					
	Light smoker	1.08	.47	1.63	<.001	1.67	<.001
	Former smoker	1.11	.15	1.88	<.001	1.85	<.001
	Never smoker	1.11	.051	1.39	<.001	1.31	<.001
**Type of registered permanent residence**							
	Urban	1 (Reference)					
	Rural	1.03	.59	0.85	<.001	0.73	<.001
**Education level**							
	Primary school and below	1 (Reference)					
	Middle school	1.35	<.001	1.42	<.001	1.40	<.001
	High school	1.74	<.001	2.16	<.001	2.34	<.001
	Associate degree and above	1.83	.004	3.08	<.001	5.06	<.001
**Ethnicity**							
	Han ethnic group	1 (Reference)					
	Ethnic minority	0.93	.33	0.78	.004	0.80	.01
**Family history of cancer**							
	Yes	1 (Reference)					
	No	0.64	<.001	0.60	<.001	0.50	<.001
	Unknown	0.47	<.001	0.48	<.001	0.38	<.001
**Occupation**							
	Tertiary industry	1 (Reference)					
	Primary industry	0.88	.03	0.84	.003	0.82	<.001
	Secondary industry	0.86	.06	0.75	<.001	0.58	<.001
	Unemployed	0.79	<.001	0.63	<.001	0.66	<.001
**Annual household income per capita (RMB)^c^**							
	<7500 RMB (<US $1117.00)	1 (Reference)					
	7500-16,667 RMB (US $1117.00-2482.28)	1.50	<.001	1.54	<.001	1.41	<.001
	16,667-30,000 RMB (US $2482.28-4468.01)	1.58	<.001	1.48	<.001	1.36	<.001
	>30,000 RMB (>US $4468.01)	1.43	<.001	1.18	.008	1.10	.12
**Administrative divisions**							
	Northwest China	1 (Reference)					
	Southwest China	0.73	.03	1.50	.02	2.18	<.001
	South China	1.06	.67	1.76	.001	2.93	<.001
	Northeast China	0.98	.89	2.12	<.001	2.62	<.001
	North China	1.17	.23	3.46	<.001	5.72	<.001
	Central China	2.25	<.001	3.79	<.001	5.28	<.001
	East China	1.24	.07	3.27	<.001	6.98	<.001

^a^The OR represents the odds ratio that higher levels of cancer literacy (level 2/level 3/level 4, compared with the reference level [level 1]) will occur given a particular exposure and the odds of the outcome occurring in the absence of that exposure.

^b^The rates were classified into 4 groups according to quartiles before the multivariate logistic regression analysis, which were level 1 (<59.46%, as the reference), level 2 (59.46%-81.08%), level 3 (81.08%-91.89%), and level 4 (>91.89%).

^c^Classified according to quartiles.

## Discussion

### Principal Results

To our knowledge, this is the first national survey to measure the level of cancer literacy through completely objective items, aiming to provide references for the Chinese government as an evaluation of former health education practices and a review of current strategies. In this work, the standardized rate of cancer literacy for the Chinese population has reached the target value proposed in Healthy China 2030 for 2022 ahead of schedule. However, from the perspective of knowledge dimensions, the corresponding rate of basic knowledge of cancer remained relatively low, and the grasp of information regarding early diagnosis and treatment also needed improvement. Furthermore, there were significant disparities in the distributions among regions and populations with different characteristics. Gender, household registration type, education level, smoking status, annual household income per capita, ethnicity, occupation, and family history of cancer were associated with cancer literacy.

### Comparison With Prior Work

Before the development of the questionnaire, the published evaluation tools for cancer literacy were systematically searched and summarized (see Table S9 in Multimedia Appendix 6 [9,10,23-34] for details). In brief, there are several established tools, such as the measure of awareness and beliefs about cancer [23] and the Cancer Awareness Measure [24]. These questionnaires contain single- or multidimensional assessments of cancer understanding, attitude, and skills and have been adopted in local survey practices. The Chinese government proposed that the current task was to raise awareness of systematic knowledge of cancer prevention and control. Accordingly, we collected key information on cancer that the general population was expected to know through a systematic review and the Delphi method [35] and formed an item pool. Existing questionnaires did not completely cover the abovementioned core knowledge, and the corresponding Chinese versions were not available or have not been validated in the Chinese population. Although there have been 3 related surveys administered during government-funded cancer screening programs in China [26-28], 2 questionnaires were not considered comprehensive, and the other multidimensional tool was developed to assess attitudes instead of knowledge, which were not suitable for a national survey on the evaluation of cancer literacy. On the other hand, the questionnaire in this study has a clear theoretical framework, comprehensive coverage of information, and acceptable reliability and validity performance, and is closely connected to the current situation in China.

Disparities among levels of cancer literacy in each dimension reflected the achievements and shortcomings of health education at this stage in China. Awareness of cancer management was relatively high, possibly because the Chinese government encouraged strengthening the professional advantages of health workers, especially in popularizing science during clinical activities [13]. Furthermore, low requirements were set for this dimension considering its strong professionalism, which might explain the better performance than in another survey [12]. The corresponding rate of cancer prevention was also acceptable [12,36], given that it was one of the main subjects in health education that was closest to daily life and would not raise concern. In general, the awareness level of primary prevention in this survey was significantly higher than a survey conducted in rural China during 2007-2014 (mean score 56.1, SD 19.3) and a Switzerland survey in 2009 (mean score 54.9, SD 23.9) [12,26], while still lower than a similar level reported by a UK study [24]. In contrast, the lower awareness of basic knowledge of cancer in China provided lessons for other developing countries. Very high proportions of the participants showed positive beliefs toward cancer outcomes in a survey conducted in 6 high-HDI countries, especially in Norway, and the main barrier to symptomatic presentation was the busy schedule [37]. However, in Chinese culture, people tend to delay diagnosis or treatment because of social stigma, fear, familial obligations, and embarrassment [38-40]. Thus, it is essential to emphasize the importance of timely interventions and address common misunderstandings through interactive communication that may affect individual choices [41,42]. Meanwhile, paying more attention to exerting the patient effect can help to eliminate misconceptions of cancer through experience sharing. In addition, there was room for improvement in skills, mainly the identification of warning symptoms [40,41,43]. A population-based survey in the United States reported awareness of an average of 8.43 (in 11) cancer symptoms, with the rates of recognition on each symptom mostly over 76% [44]. Another survey in developed countries also reported better awareness of warning symptoms, ranging from 70.09% (7.71 in 11, Sweden) to 79.09% (8.70 in 11, Canada) [37]. There are currently 4 cancer screening programs targeting different regions and populations in China [45]. During community mobilization and the screening process, health education carried out by community health workers and clinicians respectively might contribute to this. It is believed that cancer screening will be familiar to a wider range of individuals with the expansion of programs and synchronous health education. Moreover, appropriate examples of the application of knowledge in practice are expected to contribute to cultivating comprehensive cancer literacy.

There were evident variations in the levels of cancer literacy among regions within China. Several prospective studies have shown potential associations between cancer literacy and HDI values or burden of cancer, while related evidence remains limited and the majority was cross-sectional studies. Specifically, low health literacy has been proven to be related to poorer use of health care services and higher mortality [8,27], which might exacerbate the disparities in health outcomes, taking into account the existing differences in cancer burdens and health resources among regions. Urban residents, individuals with higher income, or those who finished compulsory education tended to have higher levels of awareness, possibly due to the more myriad ways that they obtain professional information and their stronger ability to absorb information [40,46]. Therefore, health education efforts should be focused on regions with lower life expectancy, years of schooling, and per capita gross national income, especially Western China. Significant disparities were also observed among populations. Populations with a family history of cancer or those older than 55 years showed acceptable levels of awareness because they might be more willing to acquire knowledge and receive synchronous education during health care [43]. This provided additional evidence to counter the stereotype that elderly individuals are not well adapted to information technologies [47]. Hence, increasing the enthusiasm of actively seeking information may improve the efficiency of health education. Participants occupied in tertiary industry showed higher cancer literacy because of higher coverage of health workers. In addition, higher awareness rates were found among nonsmokers, which was consistent with previous findings [48]. The gap in rates between genders was not distinct, consistent with the results of a national survey on health literacy [49].

### Strength and Limitations

In comparison with previous surveys focusing on single types of cancer (mainly colorectal cancer [40] and cervical cancer [43,50]) or specific populations (such as students [51], females [43,46], and elderly people [40]), this study was the first large-scale cross-sectional survey that covered almost 70% of provinces in mainland China and provided more comprehensive information. With the aim of assessing government work, this survey informs current disparities in cancer literacy in China and essential references for improvements in health education and promotion. However, there were also several limitations in this work. First, because of the limitation of data accessibility, only the distributions of the most important variables were adjusted. As it was impossible to fully consider all possible factors and approach the census population, the reported cancer literacy might be overestimated or underestimated in this study. Second, this was a cross-sectional survey, which can indicate correlations but cannot infer causality between awareness and other variables. Third, it was not appropriate to compare the overall cancer literacy with that reported in other surveys directly, considering the differences in the applied questionnaires and the main purposes. However, as the questionnaire was developed based on published articles, the items included in each dimension were consistent with the commonly recognized knowledge, and comparisons on some specific aspects can be made with caution.

### Conclusions

In summary, the weighted cancer literacy for Chinese residents was 70.05% in this cross-sectional survey. The awareness level was highest in the dimension of cancer management but was lowest for basic knowledge of cancer and the knowledge of early diagnosis and treatment. There were significant geographic, demographic, and socioeconomic disparities regarding cancer literacy, which calls for urgent efforts to carry out targeted health education for individuals with a heavier disease burden, lower income, or limited education level, especially in Southwest and Northwest China. Policy makers should conduct regular surveys to grasp the most current situations, address weaknesses in knowledge, correct misunderstandings, and move toward the goal of Healthy China 2030.

## Appendix

Multimedia Appendix 1 eMethods.

Multimedia Appendix 2 Applied questionnaire.

Multimedia Appendix 3 Response rate and baseline characteristics of enrolled participants.

Multimedia Appendix 4 Performance of content validity evaluation and item response theory (IRT) analysis of the questionnaire.

Multimedia Appendix 5 The levels of cancer literacy by administrative divisions.

Multimedia Appendix 6 Summary of key information of the published evaluation tools for cancer literacy.

## Abbreviations

HDI: Human Development Index
IRT: item response theory
PPS: probability proportional to size sampling
SSU: secondary sampling unit

## Data Availability

The study group welcomes potential collaboration to maximize the use of data. A data dictionary, a detailed study protocol, and the R programs can be reached by contacting the corresponding author of this paper. Due to Chinese legal restrictions and the current ethical approval for the study, data are not publicly available to share, but the research group can provide descriptive data in a table form. Requests can be made to WC or JH.
