# Supplementary material for: Geographic, Demographic, and Socioeconomic Disparities and Factors Associated With Cancer Literacy in China: National Cross-sectional Study
Source: JMIR Public Health Surveill. 2023 Feb 17;9:e43541. doi: 10.2196/43541 (PMC9985002; doi:10.2196/43541)
Supplement: Multimedia Appendix 1 [file publichealth_v9i1e43541_app1.docx]

**Multimedia Appendix 1: eMethods**

**Calculation of sample size:** A national cross-sectional survey was administered in seven administrative divisions of mainland China using the multistage probability proportional to size sampling (PPS) method in 2021. Local residents aged 15-74 were selected, except those registered in a collective residence (such as soldiers). The Chinese government set a target of 70 percent for cancer literacy in 2022, based on which the sample size was estimated as follows:

$$N=\frac{\mu_{\alpha}^{2}\times p\times(1-p)\times deff}{\delta^{2}}$$

In this formula, the two-sided α was set as 0.05. Design effect (deff) was recommended to be considered in the sample size of complex sampling,^1-3^ and was set as three in this study. The margin of error ($\delta$) was set as 0.05.^4^ After being divided into 2 tiers by the type of registered permanent residence, and taking into account the rates of invalid questionnaires and rejection at 10%, at least 2,152 samples had to be included, which refers to the minimum sample size required by each province to ensure their evaluations of cancer literacy.

**Sampling method:**

A 6-stages sampling process was applied in this national survey. There was a total of 31 provinces in Mainland China, divided into seven administrative divisions. We selected 21 provinces using Probability Proportionate to Size Sampling (PPS) method based on the reported population aged 15-74 in each province, with an average of 3 provinces in each administrative division. In order to ensure the representativeness of data, at least two provinces should be included in each administrative division. The following stages were conducted in the 21 provinces, respectively. The whole survey was conducted in urban and rural areas, which were classified according to the document issued by the Ministry of Civil Affairs.

First, the total number of secondary sampling units (SSUs) to be selected was allocated to each province based on their population aged 15-74 in the *2020 China Statistical Yearbook* ^5^. Then, the exact number of SSUs for each layer was further determined by the urban-rural population ratio. Due to the large population base and regional imbalance, we set a size range for SSUs in advance to ensure the homogenization of the survey in each province. Namely, the provinces were divided into 5 groups according to the size of the population aged 15-74: ≤50, 50-75, 75-100, 100-125 and >125 (million) and were assigned 8, 10, 12, 14 and 16 SSUs, respectively.

Second, the PPS method was applied to select 3 streets (for the urban layer) or towns (for the rural layer) in each SSU. In this step, the researchers listed firstly the name, divisions code and population aged 15-74 of each street. Then, each street was given a series of numbers based on the eligible population, and three random numbers in the range of 1 to the total population were generated by the computer. The street corresponding to the number range where each random number was located was finally selected.

Three-tiered sampling units were extracted in similar ways, including 2 neighborhood or village committees. Fourth, a simple random sample of 50 households was taken based on the electronic geographic listings of community households. One eligible family member in the sampled households was identified with the Kish grid method and selected to finish a questionnaire survey^6^. Thus, the provinces with the smallest sample sizes were 2,400, which was expected to meet the minimum standard.

^#^ Notes: In China, the age of population who completed the compulsory education is 15 years old, which is generally considered to have developed an independent cognition.^7^ We set 15 years old as the starting age of this survey, which is also consistent with the starting age of the national health literacy survey in China.^8^ Sixty/sixty-five years old and above are considered as the elderly, and with the increase of age, the incidence of cognitive impairment increases.^9^ Since the life expectancy of the Chinese population has been close to 78 years,^10^ we set the ending age at 74 years to ensure the completion of the survey, which is also the cutoff age recommended for some cancer screening.^11, 12^

**Quality control:**

To ensure the standardization and accuracy of this study, investigators were well trained for household surveys. Before the formal one-to-one survey, the respondents were briefed on the purpose, significance and confidentiality of this investigation in detail and signed informed consent forms. The questionnaires from every county-level site were input into EpiData (Version 3.1) software and checked in parallel on the same day as the investigation, and the data were encrypted and transmitted to the corresponding provincial unit when completed. If there was no error in 5% of the random samples, the questionnaires were uploaded to the National Cancer Center for data verification and analysis.

**Data weighting and calculation:**

The aim of this process was to adjust the distributions of the main variables in the survey sample to match *Statistics on the 2010 Population Census of the People’s Republic of China* ^13^, which represents the newest and most realistic distributions.

The key variables were selected by the research team after discussion in advance, which were sex, type of registered permanent residence, and education level. Due to data availability, three variables at most can be considered, and the above were commonly regarded as the most likely factors. Both the sample and census population were first classified into 16 layers by sex (male, female), type of registered permanent residence (urban, rural), and education level (incomplete compulsory education, junior high school, high school, college and above), and then the proportions of each layer for every province were adjusted, during which weight 1 was developed (one of the non-representativeness weights).

Similarly, the population distributions of each administrative division were adjusted by weight 2 due to the discrepant compliance among provinces with the sampling protocol (the other one of the non-representativeness weights). It was assumed that the weighted data were basically representative of each administrative division after the former two steps, and hence, the national awareness rate could be regarded as a weighted mean. Weight 3 referred to the proportions of population in each administrative divisions among China. The coefficients applied in calculations of cancer literacy varied for the provincial, district and national levels, which were weight 1, (weight 1* weight 2), and (weight 1* weight 2 *weight 3), respectively.

^#^ Notes: The provinces included in each administrative division were determined following the government document of China, and the specific rules were as follows: (1)East China: Shanghai, Jiangsu, Zhejiang, Anhui, Jiangxi, Shandong, Fujian, and Taiwan; (2)Central China: Henan, Hubei, and Hunan; (3)North China: Beijing, Tianjin, Hebei, Shanxi, and Neimenggu; (4)Northeast China: Heilongjiang, Jilin, and Liaoning; (5)South China: Guangdong, Guangxi, Hainan, Hongkong, and Macau; (6)Southwest China: Chongqing, Sichuan, Guizhou, Yunnan, and Tibet; (7)Northwest China: Shaanxi, Gansu, Qinghai, Xinjiang, and Ningxia. (Data from provinces with underlines were not available in this survey)
