# Supplementary material for: Geographic, Demographic, and Socioeconomic Disparities and Factors Associated With Cancer Literacy in China: National Cross-sectional Study
Source: JMIR Public Health Surveill. 2023 Feb 17;9:e43541. doi: 10.2196/43541 (PMC9985002; doi:10.2196/43541)
Supplement: Multimedia Appendix 2 [file publichealth_v9i1e43541_app2.docx]

**Multimedia Appendix 2:** **applied questionnaire**

**National survey on the rates of cancer literacy in China**

**Section 1: True or false questions**

**Directions: In this section, there are 13 questions. You should decide on the only one correct choice and fill in the brackets with T (for TRUE) or F (for FALSE). If you have no idea, please fill in the brackets with D (for Don't know).**

**A01** Cancer usually takes many decades to develop. ( )

**A02** By taking comprehensive measures of cancer prevention and control, some areas of China have seen a decreasing incidence and deaths of cancer. ( )

**A03** In addition to surgical treatment, cancer treatment include radiotherapy, chemotherapy, targeted therapy, immune therapy, endocrine therapy, as well as the combination of traditional Chinese and Western medicine, etc. ( )

**A04** Patients only need to actively strengthen the natural immunity during the rehabilitation of cancer, because psychological rehabilitation cannot help to maintain the stability of patient’s disease. ( )

**A05** Vaccination (such as HPV vaccine) can prevent some cancers. ( )

**A06** We can directly use the treatment which showed good curative effects on others, regardless of the pathological subtype and stage of cancer. ( )

**A07** Some cancers (such as breast cancer, colon cancer, etc.) have certain heritability, and the individuals with family of cancer should pay more attention to their health. ( )

**A08** Cancer screening is a special physical examination for cancer based on the actual situation of individuals, which has been able to detect some common types of cancers at an early stage. ( )

**A09** If someone has a persistent dry cough, finds blood in sputum, or has other symptoms, he/she should go to the hospital for further examination of lung cancer ( )

**A10** If the mole on your body becomes darker and larger in a short time, you can excise it by yourself or chose to wait for further development, while don’t have to go to the hospital immediately. ( )

**A11** Many cancers can be cured by folk prescription, taking health care products, or following the treatment plan in the advertisement. ( )

**A12** Cancer is not an incurable disease, and some cancer patients are able to survival for decades. ( )

**A13** People suffering from some chronic diseases (such as chronic hepatitis B, ulcerative colitis, etc.) have a higher risk of cancer than the general population. ( )

**Section 2: Single choice question**

**Directions: In this section, there are 13 questions. For each of them there are four choices marked A, B, C and D. You should decide on the only one correct choice and mark the corresponding letter with “√”. If you have no idea, please choose D.**

**B01 Which of the following statement is true about the relationship between cancer and lifestyle? C**

A Lifestyle determines the risk of cancer, so individuals who are heavy smoker or alcoholics would be diagnosed with cancer sooner or later.

B Lifestyle has no relationship with cancer.

C Lifestyle is closely associated with cancer, and an unhealthy lifestyle increases the risk of cancer.

D Don’t know.

**B02 Which is the most common cancer in China currently? A**

A Lung cancer.

B Bladder cancer.

C Pancreatic cancer.

D Don’t know.

**B03 Which of the following statement is true about the trend of cancer incidence in China in recent years? A**

A The incidences of lung cancer, breast cancer and colorectal cancer are increasing.

B The incidences of lung cancer, breast cancer and colorectal cancer are decreasing.

C The incidences of lung cancer, breast cancer and colorectal cancer remain constant.

D Don’t know.

**B04 Which of the following statement is true about the prevention of cancer? B**

A The occurrence of cancer is only related to a person’s health status, regardless of his/her age.

B Cancer could be occurred at any age, so we should develop a healthy lifestyle from an early age.

C Middle-aged and elderly people show high cancer incidence, so there is no need for teenagers to worry about getting cancer.

D Don’t know.

**B05 Which of the following statement is true about cancer? C**

A It’s mainly related to genetic factors.

B It’s mainly related to lifestyle.

C It’s related to both individual factors and environmental factors.

D Don’t know.

**B06 Which of the following statement is true about cancer? C**

A All types of cancer are contagious so we should keep away from cancer patients.

B Some types of cancer are contagious. For example, there are whole families who get cancer.

C Cancer itself is not contagious, but bacteria and virus relating to cancer are contagious.

D Don’t know.

**B07 Which of the following statement is true about early detection of cancers? A**

A You should choose professional medical institutions and appropriate physical examination.

B You can choose any physical examination according to your own ideas and interests.

C There is no need to take cancer examinations if you have done a general physical examination.

D Don’t know.

**B08 Which of the following statement is true about the frequency of taking cancer examinations? B**

A A one-off cancer examination is enough because cancer develops slowly.

B The interval period of cancer examinations is dependent on your age and previous diagnosis of your physical examinations.

C It’s unnecessary to take cancer examinations unless you do not feel well.

D Don’t know.

**B09 If frequent toileting occurs recently, and the stool gets bloody and thin , you should go to the hospital to further exam for: B**

A liver cancer.

B Colorectal cancer.

C Pancreatic cancer.

D Don’t know.

**B10 Which of the following statement is true about the survival of cancer? A**

A The earlier cancer is detected, the better the therapeutic efficacy is, and the longer the survival time is.

B The survival time is related to the malignant degree of the tumor, but not to the time of diagnosis.

C As long as someone is diagnosed with cancer, his/her survival time will not be too long even diagnosed at an early stage.

D Don’t know.

**B11 Which of the following statement is true about cancer examinations? B**

A It must be cancer as long as the indicators show an abnormal value during cancer examinations.

B Further diagnosis and treatment are needed if the indicators show an abnormal value during cancer examinations.

C There is no need to concern about abnormal indicators if you feel well.

D Don’t know.

**B12 Which of the following statement is true about anticancer drugs? C**

A As long as you take it, the therapeutic efficacy remains constant. You can crush it yourself or mix it with other foods.

B If you forget to take or take fewer tablets of anti-cancer drugs at one time, you should increase your dosage at the next time .

C Taking medicine should follow the doctor's advice because the therapeutic efficacy will be different when taken before and after meals.

D Don’t know.

**B13 Which of the following statement is true about reexamination? C**

A The more frequent and more comprehensive the reexamination is, the better the prognosis will be.

B As long as you take medicine on time, you don't need to go to the hospital for further consultation to avoid increasing the medical burden

C Reexamination regularly is necessary so as to detect and treat metastatic tumor and reoccurrence timely.

D Don’t know.

**Section 3: Multiple choice question**

**Directions: In this section, there are 11 questions. In this section, each question has two or more correct answers, you should decide on the correct choices and mark the corresponding letters with “√”. If you have no idea, please select E.**

**C01 What of the followings are associated with the cancer? ABCD**

A Chemical factor, such as toxic organic substances.

B Physical factors, such as radiation.

C Psychological factors, such as excessive stress and mental tension.

D Behavioral factors, such as an unhealthy lifestyle.

E Don’t know.

**C02 Which of the following biological factors can increase the risk of cancer? BCD**

A Probiotics in the gut can increase the risk of bowel cancer.

B Infection with Helicobacter pylori can increase the risk of stomach cancer.

C Infection with hepatitis B virus (HBV) can increase the risk of liver cancer.

D Infection with human papillomavirus (HPV) can increase the risk of cervical cancer.

E Don’t know.

**C03 Which of the following unhealthy lifestyle habits can increase the risk of cancer? ABD**

A Lack of exercise.

B Smoking, drinking.

C Excessive attention to personal hygiene.

D Irregular diet habits.

E Don’t know.

**C04 To prevent the development of cancer, you should do: ABC**

A Keep yourself in a good mood.

B Moderate exercise.

C Quit smoking and limit alcohol intake.

D Arrange meals according to your personal preference.

E Don’t know.

**C05 Which of the following measures can effectively reduce the incidence and mortality of cancer? ABCD**

A regular cancer examinations.

B Participate in cancer screening.

C Receive health education, and improve health literacy.

D Treat chronic infections actively (such as chronic hepatitis B).

E Don’t know.

**C06 Which of the following statements is correct about cancer prevention and treatment: ABD**

A Some cancers are completely preventable by taking active measures.

B Some cancers can be cured with the current level of medical care.

C Cancer is developing so fast that cannot be cured even if it is detected early.

D Some cancers are incurable but can be treated to relieve suffering and prolong life.

E Don’t know.

**C07 Which of the following statements is true about cancer screening: ABCD**

A Early gastrointestinal cancer can be detected by endoscopy.

B Early lung cancer can be detected by LDCT of the chest.

C Early liver cancer can be detected by abdominal B-mode ultrasound combined with alpha-fetoprotein examination.

D Early breast cancer can be detected by breast ultrasound combined with mammography.

E Don’t know.

**C08 Which of the following symptoms suggests breast cancer: BCD**

A Physical examination suggests hyperplasia of mammary glands.

B Irregular lumps in the breast.

C Nipple discharge or nipple depression and retraction.

D Orange peel-like appearance of the breast.

E Don’t know.

**C09 Which of the following symptoms may be a warning sign of cancer and should be noted: ABCD**

A Persistent hoarseness, and dry cough.

B Apparent weight loss for unknown reasons.

C Recurrent fever and weakness of unknown reasons.

D Unexplained bleeding such as blood in urine and stool.

E Don’t know.

**C10 Which of the following groups of people are at high risk of cancer? ABCD**

A Obese people.

B People aged 50 and over.

C People who are introverted and sulky.

D People who have a first-degree relative diagnosed with cancer.

E Don’t know.

**C11 Which of the following statement is correct about pain in cancer patients: AB**

A Cancer pain is one of the common symptoms of cancer patients, but most can be relieved by treatment.

B If there is cancer pain, you should follow the doctor's recommendations to relieve the pain.

C Pain occurs whenever there is cancer and there is no way to relieve it.

D Once a cancer patient has pain, it means that he/she is in the advanced stages.

E Don’t know.

**C12 For a better recovery, cancer patients should do: ABC**

A Take drugs reasonably, deal with pain and control the disease actively.

B Have a balanced diet and receive nutritional support if necessary.

C Regular reexamination, timely detection of lesions and receive interventions.

D Stay in bed for long periods of time, conserve energy, and refuse any exercise.

E Don’t know.

**Section 4:** **Basic information**

**D01.** **Gender:** ① male ② female

**D02. Date of birth:** ________

**D03.** **Your** **ethnicity:**

①The Han ② The Zhuang ③ The Hui ④ The Manchu ⑤ The Uygur ⑥ The Miao ⑦ The Yi ⑧ The Tujia ⑨ Other

**D04. Your marital status is:**

①Unmarried ② Married ③ Separated ④ Divorced ⑤ Widowed

**D05. Your educational level:**

① Never received formal education

② Graduated from primary school

③ Graduated from junior high school

④ Graduated from senior high School / technical secondary school / technical school

⑤ Graduated from college

⑥ Bachelor degree

⑦ Master degree and above

**D06. Your occupation is:**

① Agricultural personnel (agriculture, forestry, animal husbandry and fisheries)

② Professional and technical personnel (teachers, lawyers, engineers, etc.)

③ Civil servants and staff of public institutions

④ Staff of factory and mining enterprises (mining, manufacturing, construction workers, etc.)

⑤ Commercial retail or service personnel (wholesale and retail merchants, catering workers, etc.)

⑥ School students

⑦ Active military personnel

⑧ Not employed

⑨ Retired persons

⑩ Medical workers (doctors, nurses, public health workers, etc.)

**D07.** **Numbers of people in your household: _____**

**D08.** **Over the past year, your annual household income was approximately ___ CNY**

**D09. Does anyone in your family (your grandparents, parents and siblings) suffer from cancer?**

① Yes ② No ③ I don't know

**D10. Do you smoke?**

① Yes, smoke daily. Average daily numbers of cigarettes_____ (please convert the number of packs)

② Yes, but not every day. Average weekly numbers of cigarettes_____ (please convert the number of packs)

③ I used to smoke, but now I quit smoking.

④ Never smoked.

**D11. Your height is____ centimeter, your weight is____ kilogram.**

**D12. Your type of household registration is:**

① Agricultural household registration ② Non-agricultural household registration

**D13. In the past year, you consider your health to be:**

① Good ② Relatively good ③ In general ④ Relatively bad ⑤ Bad

This is the end of the investigation.

Thank you again for your support and cooperation!
