# Supplementary material for: Geographic, Demographic, and Socioeconomic Disparities and Factors Associated With Cancer Literacy in China: National Cross-sectional Study
Source: JMIR Public Health Surveill. 2023 Feb 17;9:e43541. doi: 10.2196/43541 (PMC9985002; doi:10.2196/43541)
Supplement: Multimedia Appendix 3 [file publichealth_v9i1e43541_app3.docx]

**Table S1. The response rate of each administrative division, and combined**

| **Administrative divisions** ^a^ | **Included sample** | **Unit**  **Non-response ^d^** | **Item**  **Non-response ^d^** | **Enrolled sample** | **Response rate (%)** |
| --- | --- | --- | --- | --- | --- |
| Central China | 13673 | 1599 | 112 | 15384 | 88.88 |
| East China ^b^ | 25549 | 216 | 1476 | 27241 | 93.79 |
| North China | 8254 | 375 | 334 | 8963 | 92.09 |
| Northeast China | 11069 | 1646 | 110 | 12825 | 86.31 |
| Northwest China ^†^ | 4459 | 176 | 74 | 4709 | 94.69 |
| South China | 10024 | 1094 | 1546 | 12664 | 79.15 |
| Southwest China | 7253 | 287 | 554 | 8094 | 89.61 |
| **Overall** | **80281** | **5393** | **4206** | **89880** | **89.32** |

Notes:

^a^ The provinces included in each administrative division were determined following the government document of China, and the specific rules were as follows: (1)East China: Shanghai, Jiangsu, Zhejiang, Anhui, Jiangxi, Shandong, Fujian, and Taiwan; (2)Central China: Henan, Hubei, and Hunan; (3)North China: Beijing, Tianjin, Hebei, Shanxi, and Neimenggu; (4)Northeast China: Heilongjiang, Jilin, and Liaoning; (5)South China: Guangdong, Guangxi, Hainan, Hongkong, and Macau; (6)Southwest China: Chongqing, Sichuan, Guizhou, Yunnan, and Tibet; (7)Northwest China: Shaanxi, Gansu, Qinghai, Xinjiang, and Ningxia. (Data from provinces with underlines were not available in this survey)

^b^ Among all the 21 provinces included in this survey, Xinjiang (in the Northwest China) and Shandong (in the East China) province did not record the information about unit non-response. Thus, we assumed that the unit-response rate in these three provinces were 100%, and the overall response rates were calculated based on information about item non-response, which might overestimate the response rates of the corresponding administrative divisions, as well as the national response rate.

^c^ Refers to the number of rejecting to participation of this survey or fail in household survey.

^d^ Refers to interruption in the survey or missing values in the questionnaire.

**Table S2. Characteristics of the participants and corresponding distributions according to statistics from the 6th Census in China**

|  | **Sociodemographic characteristics** | **Sample size** | **Proportions in this survey** | **Proportions in the 6^th^ Census ^a^** |
| --- | --- | --- | --- | --- |
| Administrative divisions | East China | 25549 | 31.82% | 29.72% |
|  | Central China | 13673 | 17.03% | 15.98% |
|  | North China | 8254 | 10.28% | 12.68% |
|  | Northeast China | 11069 | 13.79% | 8.73% |
|  | South China | 10024 | 12.49% | 11.69% |
|  | Southwest China | 7253 | 9.03% | 13.95% |
|  | Northwest China | 4459 | 5.55% | 7.24% |
| Gender | Male | 34127 | 42.51% | 50.86% |
|  | Female | 46154 | 57.49% | 49.14% |
| Education level ^b^ | Primary school and below | 12778 | 15.92% | 33.75% |
|  | Middle school | 19827 | 24.70% | 41.70% |
|  | High school | 16231 | 20.22% | 15.02% |
|  | Associate degree and above | 31445 | 39.17% | 9.53% |
| Type of registered permanent residence | Urban | 41138 | 51.24% | 52.14% |
|  | Rural | 39143 | 48.76% | 47.86% |
| Age, years | 15-24 | 5292 | 6.59% | 21.31% |
|  | 25-34 | 15183 | 18.91% | 18.58% |
|  | 35-44 | 17915 | 22.32% | 22.76% |
|  | 45-54 | 19179 | 23.89% | 17.28% |
|  | 55-64 | 14146 | 17.62% | 13.12% |
|  | 65-74 | 8566 | 10.67% | 6.95% |
| Ethnicity | Han ethnic group | 74375 | 92.64% | 92.10% |
|  | Ethnic minority | 5857 | 7.30% | 7.90% |
| Marital status ^c^ | Unmarried | 10855 | 13.52% | 21.60% |
|  | Married | 65112 | 81.11% | 71.33% ^d^ |
|  | Separated | 333 | 0.41% | 71.33% ^d^ |
|  | Divorced | 1954 | 2.43% | 1.38% |
|  | Widowed | 1673 | 2.08% | 5.69% |
| Occupation | Primary industry | 24471 | 30.81% | .. |
|  | Secondary industry | 4919 | 6.19% | .. |
|  | Tertiary industry | 35220 | 44.34% | .. |
|  | Unemployed | 14828 | 18.67% | .. |
| Smoking status | Daily smoker | 12187 | 15.18% | .. |
|  | Light smoker | 2563 | 3.19% | .. |
|  | Former smoker | 5282 | 6.58% | .. |
|  | Never smoker | 60012 | 74.75% | .. |

**Table S2. Characteristics of the participants and corresponding distributions according to statistics from the 6th Census in China (Continued)**

|  | **Sociodemographic characteristics** | **Sample size** | **Proportions in this survey** | **Proportions in the 6^th^ Census ^a^** |
| --- | --- | --- | --- | --- |
| Self-reported health status | Good | 24216 | 30.16% | .. |
|  | Relatively good | 29131 | 36.29% | .. |
|  | Average | 23736 | 29.57% | .. |
|  | Relatively poor | 2631 | 3.28% | .. |
|  | Poor | 549 | 0.68% | .. |
| Family history of cancer | Yes | 18129 | 22.58% | .. |
|  | No | 54989 | 68.50% | .. |
|  | Unknown | 6801 | 8.47% | .. |
| Annual household income per capita (RMB) ^e^ | <7500 | 15198 | 22.80% | .. |
|  | 7500-16667 | 21313 | 31.98% | .. |
|  | 1667-30000 | 12817 | 19.23% | .. |
|  | >30000 | 17323 | 25.99% | .. |

Notes:

^a^ Data were extracted from *Statistics on the 2010 Population Census of the People’s Republic of China (*[*http://www.stats.gov.cn/tjsj/pcsj/rkpc/6rp/indexch.htm*](http://www.stats.gov.cn/tjsj/pcsj/rkpc/6rp/indexch.htm)*)*. This study focused on the population aged 15-74 years.

^b^ Data used for the calculation of “Proportions in the 6th Census” were based on populations aged 6 years and above, limited by data accessibility.

^c^ Data used for the calculation of “Proportions in the 6th Census” were based on populations aged 15 years and above, limited by data accessibility.

^d^ The proportions of both married and separated individuals.

^e^ Classified according to quartiles.
