# Supplementary material for: Geographic, Demographic, and Socioeconomic Disparities and Factors Associated With Cancer Literacy in China: National Cross-sectional Study
Source: JMIR Public Health Surveill. 2023 Feb 17;9:e43541. doi: 10.2196/43541 (PMC9985002; doi:10.2196/43541)
Supplement: Multimedia Appendix 4 [file publichealth_v9i1e43541_app4.docx]

**Table S3. Performance of content validity evaluation on the primary indicators based on expert grading**

| **Primary indicators** | **Importance** | | **correlation** | | **Weight** | |
| --- | --- | --- | --- | --- | --- | --- |
|  | **Mean ± SD** | **CV** | **Mean ± SD** | **CV** | **Mean ± SD** | **CV** |
| 1 Basic sense of cancer | 4.73±0.61 | 0.13 | 4.71±0.54 | 0.12 | 18.60±5.74 | 0.31 |
| 2 Cancer prevention | 4.90±0.31 | 0.06 | 4.81±0.39 | 0.08 | 25.98±6.81 | 0.26 |
| 3 Early detection and intervention | 4.92±0.28 | 0.06 | 4.83±0.48 | 0.10 | 26.56±6.72 | 0.25 |
| 4 Cancer treatment | 4.54±0.62 | 0.14 | 4.52±0.65 | 0.14 | 16.56±5.97 | 0.36 |
| 5 Patients’ recovery | 4.46±0.74 | 0.17 | 4.42±0.74 | 0.17 | 12.29±6.35 | 0.52 |

Notes: CV, coefficient of variation

Performance of content validity was evaluated by expert in three aspects, which were (1) the significance in the prevention and control of cancer for each domain/item (Significance, with the grade ranging from 1 to 5), (2) the association between the domain/item and the topic of the questionnaire/Cancer Literacy (Correlation, with the grade ranging from 1 to 5), and (3) the weight you would like to allocate for the domain/item in the questionnaire (Weight, with the grade ranging from 0 to 100, determining the number of related items in the final questionnaire), respectively.

**Table S4. Performance of content validity evaluation on the secondary indicators based on expert grading**

| **Primary indicators** | **Secondary indicators** | **Importance** | | **correlation** | | **Weight** | |
| --- | --- | --- | --- | --- | --- | --- | --- |
|  |  | **Mean ± SD** | **CV** | **Mean ± SD** | **CV** | **Mean ± SD** | **CV** |
| 1 Basic sense of cancer | 1.1 Attitudes and beliefs | 4.71±0.65 | 0.14 | 4.73±0.57 | 0.12 | 45.08±13.69 | 0.30 |
|  | 1.2 Basic knowledge | 4.75±0.53 | 0.11 | 4.71±0.54 | 0.12 | 54.92±13.69 | 0.25 |
| 2 Cancer prevention | 2.1 Risk factors | 4.90±0.31 | 0.06 | 4.90±0.31 | 0.06 | 46.38±8.31 | 0.18 |
|  | 2.2 Prevention measures | 4.90±0.31 | 0.06 | 4.88±0.33 | 0.07 | 53.63±8.31 | 0.15 |
| 3 Early detection and intervention | 3.1 Significance of early detection | 4.81±0.45 | 0.09 | 4.77±0.59 | 0.12 | 24.40±8.00 | 0.33 |
|  | 3.2 Identification of warning symptoms | 4.81±0.39 | 0.08 | 4.77±0.42 | 0.09 | 25.25±8.88 | 0.35 |
|  | 3.3 Early diagnosis of cancer | 4.90±00.31 | 0.06 | 4.81±0.49 | 0.10 | 28.33±6.32 | 0.22 |
|  | 3.4 Early treatment of cancer | 4.88±0.39 | 0.08 | 4.81±0.49 | 0.10 | 22.02±7.68 | 0.35 |
| 4 Cancer treatment | 4.1 Standardized treatment | 4.79±0.50 | 0.11 | 4.75±0.56 | 0.12 | 41.73±9.00 | 0.22 |
|  | 4.2 Regular check | 4.81±0.45 | 0.09 | 4.75±0.56 | 0.12 | 29.83±8.41 | 0.28 |
|  | 4.3 Main treatment of cancer | 4.46±0.85 | 0.19 | 4.27±0.96 | 0.23 | 28.44±8.72 | 0.31 |
| 5 Patients’ recovery | 5.1 Psychological rehabilitation | 4.63±0.64 | 0.14 | 4.63±0.67 | 0.15 | 49.60±8.77 | 0.18 |
|  | 5.2 Physiological rehabilitation | 4.56±0.68 | 0.15 | 4.52±0.71 | 0.16 | 50.40±8.77 | 0.17 |

Notes: CV, coefficient of variation

Performance of content validity was evaluated by expert in three aspects, which were (1) the significance in the prevention and control of cancer for each domain/item (Significance, with the grade ranging from 1 to 5), (2) the association between the domain/item and the topic of the questionnaire/Cancer Literacy (Correlation, with the grade ranging from 1 to 5), and (3) the weight you would like to allocate for the domain/item in the questionnaire (Weight, with the grade ranging from 0 to 100, determining the number of related items in the final questionnaire), respectively.

**Table S5. Performance of content validity evaluation on the tertiary indicators based on expert grading**

| **Primary indicators** | **Secondary indicators** | **Tertiary indicators** | **Importance** | | **Sensitivity** | | **Specificity** | | **Practicality** | | **Weight (%)** | |
| --- | --- | --- | --- | --- | --- | --- | --- | --- | --- | --- | --- | --- |
|  |  |  | **Mean ± SD** | **CV** | **Mean ± SD** | **CV** | **Mean ± SD** | **CV** | **Mean ± SD** | **CV** | **Mean ± SD** | **CV** |
| 1 Basic sense of cancer | 1.1 Attitudes and beliefs | 1.1.1 Cancer is a chronic disease | 4.48±0.71 | 0.16 | 3.88±0.93 | 0.24 | 4.16±0.69 | 0.17 | 4.64±0.57 | 0.12 | 17.88±6.60 | 0.37 |
|  |  | 1.1.2 Cancer is preventable and treatable | 4.92±0.28 | 0.06 | 4.40±0.65 | 0.15 | 4.28±0.68 | 0.16 | 4.28±0.68 | 0.16 | 24.28±7.64 | 0.31 |
|  |  | 1.1.3 Cancer is closely related to life-styles | 4.72±0.54 | 0.11 | 4.28±0.68 | 0.16 | 4.12±0.60 | 0.15 | 4.36±0.76 | 0.17 | 21.48±6.31 | 0.29 |
|  |  | 1.1.4 Cancer is not contagious | 4.08±1.04 | 0.25 | 4.00±1.00 | 0.25 | 3.76±0.83 | 0.22 | 4.20±0.82 | 0.19 | 13.08±4.62 | 0.35 |
|  |  | 1.1.5 Humans can coexist with cancer | 4.20±0.82 | 0.19 | 3.96±0.89 | 0.22 | 3.84±0.85 | 0.22 | 4.12±0.73 | 0.18 | 12.28±5.28 | 0.43 |
|  |  | 1.1.6 Recognize the limitations of Medicine | 4.20±0.87 | 0.21 | 3.64±0.86 | 0.24 | 3.60±0.71 | 0.20 | 3.72±0.74 | 0.20 | 11.28±5.33 | 0.47 |
|  | 1.2 Basic knowledge | 1.2.1 Epidemiological knowledge | 4.24±0.88 | 0.21 | 4.04±0.84 | 0.21 | 4.24±0.66 | 0.16 | 4.12±0.73 | 0.18 | 47.20±9.36 | 0.20 |
|  |  | 1.2.2 Etiological knowledge | 4.68±0.69 | 0.15 | 4.32±0.85 | 0.20 | 4.32±0.63 | 0.15 | 4.08±0.76 | 0.19 | 53.20±9.88 | 0.19 |
| 2 Cancer prevention | 2.1 Risk factors | 2.1.1 Family history | 4.36±0.76 | 0.17 | 4.20±0.58 | 0.14 | 4.12±0.60 | 0.15 | 4.28±0.98 | 0.23 | 17.20±4.35 | 0.25 |
|  |  | 2.1.2 Occupational exposures | 4.40±0.65 | 0.15 | 4.16±0.90 | 0.22 | 4.04±0.93 | 0.23 | 4.16±1.03 | 0.25 | 17.00±4.56 | 0.27 |
|  |  | 2.1.3 Unhealthy life-styles | 4.88±0.33 | 0.07 | 4.48±0.59 | 0.13 | 4.24±0.78 | 0.18 | 4.48±0.65 | 0.15 | 22.00±5.20 | 0.24 |
|  |  | 2.1.4 Environmental factors | 4.56±0.65 | 0.14 | 4.08±0.70 | 0.17 | 4.00±0.71 | 0.18 | 3.92±0.76 | 0.19 | 16.60±4.94 | 0.30 |
|  |  | 2.1.5 Infection history | 4.40±0.76 | 0.17 | 4.20±0.71 | 0.17 | 4.20±0.76 | 0.18 | 4.08±1.00 | 0.25 | 13.92±5.12 | 0.37 |
|  |  | 2.1.6 Disease history | 4.20±0.76 | 0.18 | 3.88±0.88 | 0.23 | 3.96±0.73 | 0.19 | 4.08±0.95 | 0.23 | 13.88±4.58 | 0.33 |
|  | 2.2 Prevention measures | 2.2.1 Avoid risk factors | 4.68±0.56 | 0.12 | 4.40±0.71 | 0.16 | 4.44±0.71 | 0.16 | 4.40±0.71 | 0.16 | 22.20±5.02 | 0.23 |
|  |  | 2.2.2 Reduce occupational exposures | 4.52±0.65 | 0.14 | 4.48±0.59 | 0.13 | 4.48±0.51 | 0.11 | 4.36±0.70 | 0.16 | 19.60±3.80 | 0.19 |
|  |  | 2.2.3 Vaccine inoculation | 4.16±0.85 | 0.20 | 4.24±0.78 | 0.18 | 4.20±0.71 | 0.17 | 4.32±0.80 | 0.19 | 18.40±6.08 | 0.33 |
|  |  | 2.2.4 Healthy life-styles | 4.84±0.37 | 0.08 | 4.28±0.61 | 0.14 | 4.24±0.66 | 0.16 | 4.32±0.63 | 0.15 | 25.20±5.86 | 0.23 |
|  |  | 2.2.5 Other prevention measures | 4.08±0.81 | 0.20 | 3.60±0.87 | 0.24 | 3.68±0.80 | 0.22 | 3.80±0.82 | 0.21 | 14.60±6.28 | 0.43 |
| 3 Early detection and intervention | 3.1 Significance of early detection | 3.1.1 Benefits of regular physical examination | 4.84±0.47 | 0.10 | 4.40±0.65 | 0.15 | 4.36±0.76 | 0.17 | 4.52±0.65 | 0.14 | 51.20±6.00 | 0.12 |
|  |  | 3.1.2 Benefits of early detection and intervention | 4.96±0.20 | 0.04 | 4.52±0.59 | 0.13 | 4.48±0.59 | 0.13 | 4.36±0.81 | 0.19 | 48.80±6.00 | 0.12 |
|  | 3.2 Identification of warning symptoms | 3.2.1 Warning signs of digestive system cancer | 4.64±0.57 | 0.12 | 4.28±0.74 | 0.17 | 4.32±0.69 | 0.16 | 4.32±0.69 | 0.16 | 29.00±5.59 | 0.19 |
|  |  | 3.2.2 Warning signs of respiratory cancer | 4.60±0.58 | 0.13 | 4.12±0.73 | 0.18 | 4.08±0.64 | 0.16 | 4.28±0.68 | 0.16 | 27.60±4.59 | 0.17 |
|  |  | 3.2.3 Warning signs of other cancer | 4.04±0.73 | 0.18 | 3.60±0.71 | 0.20 | 3.64±0.70 | 0.19 | 3.84±0.69 | 0.18 | 21.60±8.26 | 0.38 |
|  | 3.3 Early diagnosis of cancer | 3.3.1 Recognize own susceptibility to disease | 4.88±0.33 | 0.07 | 4.28±0.54 | 0.13 | 4.36±0.64 | 0.15 | 4.36±0.57 | 0.13 | 46.80±7.48 | 0.16 |
|  |  | 3.3.2 Take targeted examination | 4.76±0.66 | 0.14 | 4.44±0.65 | 0.15 | 4.52±0.65 | 0.14 | 4.28±0.89 | 0.21 | 53.20±7.48 | 0.14 |
|  | 3.4 Early treatment of cancer | 3.4.1 Receive treatment timely | 4.72±0.61 | 0.13 | 4.36±0.76 | 0.17 | 4.24±0.83 | 0.20 | 4.40±0.71 | 0.16 | 100.00 | 0.00 |
| 4 Cancer treatment | 4.1 Standardized treatment | 4.1.1 Follow the doctor's advice | 4.84±0.47 | 0.10 | 4.48±0.65 | 0.15 | 4.32±0.90 | 0.21 | 4.60±0.58 | 0.13 | 54.92±15.92 | 0.29 |
|  |  | 4.1.2 Not superstitious, and don't believe in folk prescription | 4.40±0.82 | 0.19 | 4.16±0.75 | 0.18 | 4.08±0.95 | 0.23 | 4.00±0.76 | 0.19 | 21.32±8.48 | 0.40 |
|  |  | 4.1.3 Visit formal medical institutions for treatment | 4.40±0.82 | 0.19 | 4.12±0.73 | 0.18 | 4.04±0.89 | 0.22 | 4.00±0.87 | 0.22 | 23.72±9.18 | 0.39 |
|  | 4.2 Regular check | 4.2.1 Take regular check | 4.80±0.50 | 0.10 | 4.56±0.65 | 0.14 | 4.48±0.71 | 0.16 | 4.64±0.64 | 0.14 | 100.00 | 0.00 |
|  | 4.3 Main treatment of cancer | 4.3.1 Surgical operation | 4.56±0.82 | 0.18 | 4.32±0.85 | 0.20 | 4.36±0.86 | 0.20 | 4.40±0.58 | 0.13 | 52.20±7.92 | 0.15 |
|  |  | 4.3.2 Non operative therapy | 4.44±0.87 | 0.20 | 4.08±0.81 | 0.20 | 4.08±0.81 | 0.20 | 4.28±0.61 | 0.14 | 47.80±7.92 | 0.17 |
| 5 Patients’ recovery | 5.1 Psycholog-ical rehabilitation | 5.1.1 Positive attitude | 4.88±0.33 | 0.07 | 4.52±0.51 | 0.11 | 4.28±0.84 | 0.20 | 4.12±0.78 | 0.19 | 40.52±10.39 | 0.26 |
|  |  | 5.1.2 Psychological adjustment skills | 4.48±0.65 | 0.15 | 4.28±0.61 | 0.14 | 3.76±0.78 | 0.21 | 3.80±0.65 | 0.17 | 30.12±6.48 | 0.22 |
|  |  | 5.1.3 Seek medical help | 4.36±0.64 | 0.15 | 4.08±0.76 | 0.19 | 3.80±0.71 | 0.19 | 4.12±0.67 | 0.16 | 28.52±10.32 | 0.36 |
|  | 5.2 Physiolog-ical rehabilitation | 5.2.1 Self rehabilitation | 4.44±0.65 | 0.15 | 4.04±0.84 | 0.21 | 4.16±0.69 | 0.17 | 4.08±0.76 | 0.19 | 32.60±7.09 | 0.22 |
|  |  | 5.2.2 Medical rehabilitation | 4.60±0.65 | 0.14 | 4.12±0.78 | 0.19 | 4.28±0.61 | 0.14 | 4.12±0.67 | 0.16 | 36.60±8.86 | 0.24 |
|  |  | 5.2.3 Pain management | 4.64±0.49 | 0.11 | 4.24±0.72 | 0.17 | 4.28±0.54 | 0.13 | 4.16±0.75 | 0.18 | 30.00±8.04 | 0.27 |

**Table S6. Item Response Theory Parameters Estimates for the applied questionnaire by item**

| **Domain** | **Item** | **a** | **b** |
| --- | --- | --- | --- |
| 1 Basic sense of cancer | A01 | 0.37 | -1.23 |
|  | A02 | 1.1 | -1.7 |
|  | A12 | 0.75 | -0.68 |
|  | B01 | 1.71 | -1.02 |
|  | B02 | 1.45 | -1.37 |
|  | B03 | 1.31 | -0.8 |
|  | B06 | 1.51 | -0.83 |
|  | C01 | 1.55 | -0.56 |
|  | C06 | 1.91 | -0.49 |
| 2 Cancer prevention | A05 | 1.13 | -1.41 |
|  | A07 | 1.07 | -1.58 |
|  | A13 | 1.18 | -1.61 |
|  | B04 | 1.26 | -1.13 |
|  | B05 | 1.48 | -1.07 |
|  | C02 | 2.38 | -0.5 |
|  | C03 | 1.91 | -0.53 |
|  | C04 | 3.11 | -1.02 |
|  | C05 | 3.67 | -0.63 |
| 3 Early detection and intervention | A08 | 1.18 | -1.8 |
|  | A09 | 0.98 | -1.37 |
|  | A10 | 1.17 | -1.11 |
|  | B07 | 1.76 | -1.08 |
|  | B09 | 1.66 | -1.2 |
|  | B10 | 1.46 | -1.03 |
|  | B11 | 1.9 | -1.18 |
|  | C07 | 2.62 | -0.43 |
|  | C08 | 2.56 | -0.41 |
|  | C09 | 1.51 | 0.07 |
| 4 Cancer treatment | A03 | 1.37 | -1.89 |
|  | A06 | 1.55 | -0.65 |
|  | A11 | 2 | -1.15 |
|  | B08 | 2.42 | -1.03 |
|  | B12 | 2.94 | -0.94 |
|  | B13 | 3.11 | -1.02 |
| 5 Patients’ recovery | A04 | 0.88 | -1.2 |
|  | C10 | 2.79 | -0.82 |
|  | C11 | 3.58 | -0.95 |
