# Supplementary material for: Geographic, Demographic, and Socioeconomic Disparities and Factors Associated With Cancer Literacy in China: National Cross-sectional Study
Source: JMIR Public Health Surveill. 2023 Feb 17;9:e43541. doi: 10.2196/43541 (PMC9985002; doi:10.2196/43541)
Supplement: Multimedia Appendix 5 [file publichealth_v9i1e43541_app5.docx]

**Table S7. The levels of cancer literacy by administrative divisions and secondary indicators**

| **Primary indicators** | **Secondary indicators** | **Administrative divisions** | | | | | | | |
| --- | --- | --- | --- | --- | --- | --- | --- | --- | --- |
|  |  | **Overall** | **Northeast China** | **North China** | **Northwest China** | **Central China** | **East**  **China** | **South China** | **Southwest China** |
| 1 Basic sense of cancer | 1.1 Attitudes and beliefs | 63.35  (62.72-63.99) | 61.88  (60.92-62.84) | 64.77  (61.90-67.65) | 52.65  (51.18-54.13) | 65.49  (64.11-66.88) | 65.37  (64.41-66.34) | 56.06  (54.55-57.57) | 53.41  (52.42-54.39) |
|  | 1.2 Basic knowledge | 71.04  (70.40-71.69) | 69.46  (68.42-70.50) | 68.56  (65.41-71.71) | 57.87  (56.28-59.47) | 72.12  (70.65-73.59) | 74.12  (73.18-75.06) | 62.81  (61.27-64.34) | 62.83  (61.81-63.85) |
| 2 Cancer prevention | 2.1 Risk factors | 69.89  (69.21-70.56) | 62.45  (61.48-63.41) | 68.63  (65.80-71.45) | 57.15  (55.63-58.67) | 68.95  (67.39-70.51) | 73.22  (72.19-74.26) | 64.64  (63.04-66.25) | 61.45  (60.41-62.49) |
|  | 2.2 Prevention measures | 71.97  (71.28-72.65) | 64.78  (63.62-65.94) | 72.91  (70.10-75.71) | 59.47  (57.82-61.11) | 72.39  (70.90-73.88) | 74.54  (73.48-75.60) | 67.34  (65.62-69.05) | 61.88  (60.80-62.96) |
| 3 Early detection and intervention | 3.1 Significance of early detection | 78.08  (77.41-78.75) | 73.20  (72.07-74.33) | 78.97  (76.58-81.36) | 64.04  (62.11-65.97) | 80.63  (79.16-82.10) | 81.25  (80.25-82.26) | 69.08  (67.12-71.04) | 63.11  (61.85-64.38) |
|  | 3.2 Identification of warning symptoms | 70.66  (69.98-71.34) | 67.65  (66.51-68.80) | 72.19  (69.90-74.49) | 54.78  (52.95-56.61) | 69.55  (68.03-71.08) | 74.50  (73.46-75.54) | 61.61  (59.70-63.52) | 58.09  (56.95-59.22) |
|  | 3.3 Early diagnosis of cancer | 59.05  (58.32-59.79) | 53.02  (51.72-54.33) | 57.66  (55.58-59.75) | 41.39  (39.74-43.04) | 59.61  (57.78-61.43) | 62.71  (61.57-63.85) | 51.56  (49.76-53.36) | 47.94  (46.74-49.14) |

**Table S7. The levels of cancer literacy by administrative divisions and secondary indicators (Continued)**

| **Primary indicators** | **Secondary indicators** | **Administrative divisions** | | | | | | | |
| --- | --- | --- | --- | --- | --- | --- | --- | --- | --- |
|  |  | **Overall** | **Northeast China** | **North China** | **Northwest China** | **Central China** | **East**  **China** | **South China** | **Southwest China** |
|  | 3.4 Early treatment of cancer | 73.16  (72.38-73.94) | 66.79  (65.46-68.13) | 75.62  (72.03-79.21) | 61.49  (59.73-63.24) | 78.59  (77.08-80.10) | 74.85  (73.69-76.01) | 66.45  (64.32-68.57) | 57.63  (56.33-58.93) |
| 4 Cancer treatment | 4.1 Standardized treatment | 71.04  (70.34-71.74) | 65.34  (64.06-66.62) | 76.20  (74.07-78.32) | 59.01  (57.24-60.78) | 76.26  (74.71-77.82) | 72.14  (71.06-73.22) | 64.53  (62.68-66.37) | 57.06  (55.89-58.23) |
|  | 4.2 Regular check | 76.22  (75.47-76.97) | 70.34  (68.96-71.71) | 81.94  (79.86-84.02) | 66.37  (64.47-68.26) | 81.23  (79.58-82.88) | 77.21  (76.06-78.36) | 70.06  (67.88-72.25) | 61.85  (60.55-63.15) |
|  | 4.3 Main treatment of cancer | 84.21  (83.38-85.03) | 82.32  (80.91-83.74) | 79.52  (75.67-83.37) | 79.56  (77.42-81.70) | 86.30  (84.61-87.98) | 86.67  (85.45-87.89) | 78.03  (75.82-80.24) | 76.05  (74.70-77.41) |
| 5 Patients’ recovery | 5.1 Psychological rehabilitation | 73.44  (72.54-14.33) | 69.20  (67.78-70.63) | 70.25  (66.71-73.80) | 59.42  (57.34-61.51) | 76.09  (74.34-77.84) | 76.11  (74.70-77.51) | 66.60  (64.37-68.84) | 65.23  (63.91-66.55) |
|  | 5.2 Physiological rehabilitation | 66.13  (65.16-67.10) | 62.59  (60.83-64.35) | 74.85  (71.89-77.80) | 48.79  (46.33-51.26) | 70.15  (67.95-72.34) | 66.60  (65.11-68.09) | 61.26  (58.69-63.83) | 52.74  (51.14-54.34) |

**Table S8. Rates of cancer literacy, Human Development Index values, and the burdens of cancer in seven main regions in China.**

|  | **Rate of cancer literacy, %** | **Human Development Index value ^a^** | **ASRs of incidence ^b^** | **ASRs of mortality ^b^** |
| --- | --- | --- | --- | --- |
| East China | 72.65 | 0.76 | 193.7 | 115.6 |
| Central China | 71.73 | 0.73 | 185.5 | 109.4 |
| North China | 70.73 | 0.76 | 213.2 | 134.5 |
| Northeast China | 65.38 | 0.75 | 189.2 | 116.4 |
| South China | 63.21 | 0.75 | 202.4 | 122.4 |
| Southwest China | 59.00 | 0.69 | 226.7 | 170.2 |
| Northwest China | 57.09 | 0.71 | 207.9 | 133.2 |

^a^ The values of the Human Development Index were calculated based on corresponding values of the included provinces, which were extracted from the National Human Development Report Special Edition, available on the website of the United Nations Development Programme (https://www.cn.undp.org/content/China/en/home/library/human_development/national-human-development-report-special-edition.html).

^b^ ASR: Age-standardized (Segi Standard Population) rates [2].
