# Supplementary material for: Geographic, Demographic, and Socioeconomic Disparities and Factors Associated With Cancer Literacy in China: National Cross-sectional Study
Source: JMIR Public Health Surveill. 2023 Feb 17;9:e43541. doi: 10.2196/43541 (PMC9985002; doi:10.2196/43541)
Supplement: Multimedia Appendix 6 [file publichealth_v9i1e43541_app6.docx]

**Tables S9 Summary of key information of the published evaluation tools for cancer cognition**

| **Basic information** | | **Questionnaire design** | | | | | | **Questionnaire evaluation** | |
| --- | --- | --- | --- | --- | --- | --- | --- | --- | --- |
| **Reference** | **Country/ Region** | **Questionnaire** | **Language** | **Target population** | **No. of dimensions** | **Contents of dimensions** | **No. of items** | **Reliability** | **Validity** |
| Su et al，2009 [25] | Taiwan，China | Cancer Knowledge Scale for Elders | NA | (1) 65 years of age or older;  (2) non institutionalised;  (3) able to communicate;  (4) no known cognitive and mental impairment;  (5) no cancer history. | 4 | general cancer knowledge（7 items）, cancer prevention（9 items）, cancer warning signs（7 items） and cancer detection behaviours（6 items） | 29 | The KR-20 coefficients were 0.87 for the total scale, 0.85 for cancer warning signs subscale, 0.79 for cancer detection subscale, 0.67 for general cancer knowledge subscale and 0.76 or cancer prevention subscale. | 1. Construct validity：the factor number and factor contents were consistent with the initial proposed conceptual structure. 2. Criterion-related validity：cancer knowledge was positively correlated with cancer-related health behaviour (r = 0.78, p < 0.001). |
| Stubbings et al，2009 [24] | UK | the Cancer Awareness Measure (CAM) | English | general public | 6 | 1. 10 items on awareness of warning signs (one open-ended question and nine recognition items);  2. nine items on anticipated time to seek medical advice (asking about each of the warning signs);  3. 10 items on barriers to seeking medical advice (covering a range of practical, service delivery and emotional barriers);  4. 13 items on awareness of risk factors (one open-ended question, 11 recognition items and one asking participants to rank the importance of different types of risk factor);  5. seven items on cancer incidence (one asking about overall cancer incidence and six asking about the three most common cancers for men and women)  6. six items on awareness of NHS screening programmes (asking about awareness of the cervical, breast and bowel screening programmes and the age from which screening is offered for each). | 55 | 1.Cronbach’s α was 0.77； 2.With the exception of incidence of common cancers, high correlations over time were found for all sections. | The cancer experts scored consistently higher than the nonmedical Academics. |
| Diviani et al，2011 [9] | Switzerland | A measure of Cancer Literacy | Italian | Ticino (Italian-speaking Switzerland) residents | 5 | cancer risk（9 items）, detection and diagnosis（11 items）, treatment（4 items）, coping with the disease（10 items）, and information（3 items） | 37 | 1.Internal consistency of the final full scale was satisfactory, with an alpha coefficient of .769. 2.Four-week test-retest reliability data for the CLS was satisfactory (r = .721, p < .001). | The PCA conducted for the total scores of the five subscales indicated two components content validity assessment showed good (>78%) agreement for all individual items for clarity and relevance. |
| Simon et al，2012 [23] | Australia, Canada, Denmark, Norway, Sweden,  the UK | Awareness and Beliefs about Cancer (ABC) | UK English，Australian English, Canadian English, Canadian French, Danish, Swedish, Norwegian | people aged 50+ in six countries (Australia, Canada, Denmark, Norway, Sweden and the UK) | 6 | the core measure included  1. awareness of cancer symptoms (1 open ‘symptom recall’ item, 11 closed‘symptom recognition’ items),  2. awareness of cancer outcomes (4 items),  3. help-seeking intentions (4 items), 4. beliefs about cancer; including beliefs about outcomes and the value of early presentation (6 items),  5. beliefs about barriers to symptomatic presentation (5 items)  6. estimated age at which people are most likely to develop cancer (1 item).  The optional modules were awareness of risk factors for cancer (13 ‘risk factor recognition’items) and beliefs and behaviour in relation to breast and colorectal screening (8 items). | 32 ‘core’ items, plus modules on cancer screening (8 items) and risk factors (13 items) | 1. The internal consistency for the aggregate symptom and risk factor scores was good (Cronbach’s α >0.70), but internal consistency was lower for the aggregate scores for barriers to symptomatic presentation (0.52) and beliefs about cancer outcomes (0.49). 2. In test–retest reliability analyses, the aggregate scores all reached ‘substantial’(≥60%) agreement between administrations. | Content validity assessment showed good (>78%) agreement for all individual items for clarity and relevance. |
| Mazor et al，2012 [29, 30] | USA | the Cancer Message Literacy Test-Listening (CMLT-Listening)  the Cancer Message Literacy Test-Reading (CMLT-Reading) | English | Healthy adults aged 40–70 who had been enrolled for at least five years, and lived or received care in reasonable proximity to the study session locations. |  | The CMLT-Listening  1. assesses comprehension of spoken messages related to cancer prevention and screening; 2. is self-administered via computer; 3. begins with a brief, computer-narrated introduction, instructions and sample items; 4. The test includes 15 spoken messages presented in video, each with 2–4 associated items (48 items total).  5. Items were developed using the sentence verification technique (SVT) and are paraphrases of video message content; participants indicate whether the item meaning is the same as the original message.  6. Test administration takes approximately 1 h.  7. No reading is required.  The CMLT-Reading  1. assesses comprehension of written messages on cancer prevention and screening.  2. It is self-administered on paper.  3. It contains 6 messages, each with 3–4 associated items (23 items total).  4. For each item, the participant must indicate whether a statement has the same meaning as the original message.  5. CMLT-Reading items were also developed using the SVT.  6. Administration time is approximately 10 min. |  | Score reliabilities were good (CMLT-Listening: alpha = .84) to adequate (CMLT-Reading: alpha = .75). | Scores on both CMLT tests were positively and significantly correlated with scores on the REALM, numeracy, cancer knowledge and the cognitive tests. |
| Loo et al，2013 [31] | Malaysia | the Cancer Awareness Questionnaire (CAQ) | English | Malaysian Undergraduate Students of Chinese Ethnicity | 4 | 1. awareness of cancer warning signs（13 items） and screening tests（15 items） ;  2. knowledge of cancer risk factors（23 items，including 19 positive statements and 4 negative statements）;  3. barriers in seeking medical advice（11 items）;  4. attitudes towards cancer and cancer prevention（15 items）. | 63 | 1. a high total internal consistency (Cronbach’s α=0.77)； 2.the test-retest reliability obtaining a correlation of 0.72 (p<0.001) overall. | satisfactory construct validity |
| Dumenci et al，2014 [32] | USA | Cancer Health Literacy Test（CHLT-30 & CHLT-6） | English Spanish | English-speaking cancer patients 18 years of age or older |  | different domains of cancer health literacy（knowledge，reading，and numeracy） | 30 | 1. The CHLT-30 has Cronbach’s alpha internal consistency reliability of .88, 2. McDonald’s omega reliability of .89； 3. 2-week test–retest reliability of .90, and 6-month test–retest reliability of .92； 4. all of which are indicative of a highly consistent measure of cancer health literacy. | 1. The CHLT-30 was a significant predictor of the outcome (p<.001).  2. The standardized path coefficient was moderately high (β=.41) linking the new instrument to health decisions as envisioned by the Institute of Medicine’s definition of health literacy |
| Diviani et al，2014 [10] | Switzerland | the Cancer Literacy Score (CLS) | Italian | Ticino residents | 5 | 37 knowledge items regarding different aspects of cancer,15 grouped in 5 subscales (cancer risk, detection and diagnosis, treatment, coping, and information) | 37 | The CL scale presented satisfactory internal consistency (Cronbach’s alpha=0.769). |  |
| Barros et al，2019 [33] | Portugal | Students’ Knowledge and Perceptions About Cancer (SKPaC) | Portuguese | adolescent students | 3 | 1. perceptions about cancer (14 items);  2. knowledge about cancer (18 items);  3. students’ socio-biographic characterization (3 items). | 35 | 1.Cronbach’s alpha values present an acceptable or good internal reliability of the scale with a total Cronbach’s alpha of 0.78 in the test and 0.819 on the retest.  2.Test-retest reliability was also good, when analyzing perceptions (0.905 vs. 0.947) and effective knowledge (0.610 vs. 0.757) in separate. | Face validity: the majority of the respondents recognized the objective of the instrument and considering it suitable to proper evaluate cancer knowledge. |
| Echeverri et al，2020 [34] | USA | Multidimensional Cancer Literacy Questionnaire (MCLQ) | Spanish, English | Inclusion criteria for the field test were: ages 25 years old or older and living in Louisiana. Recruitment was stratified to obtain similar numbers by race (Latinos, African American and Whites) and gender (male/female). | 3 | 1. Facilitators Domain：5 factors (28 items) Motivation to screen (7 items)，Access to information (8 items)，Intention to screen (5 items)，Trust in physicians (4 items)，Preferences about providers (4 items) 2. Barriers Domain：7 factors (26 items) Lack awareness (2 items)，Personal discomfort (5 items)，Impediments to screen (6 items)，Lack of resources (5 items)，Poor English skills (3 items)，Communication problems (3 items)，Low locus of control (2 items) 3. Cultural Domain：8 factors (28 items) Stigmas about cancer (2 items)，Fatalistic attitude (3 items)，Beliefs about treatment (2 items)，Beliefs about prevention (5 items)，Self-determination (4 items)，Perceived cancer risk (4 items)，Worriedness about cancer (4 items)，Symptomatic deterrents (4 items) | 82 | Cronbach alpha for the scale score was 0.89 and internal consistency reliability coeffcients for each factor were all above 0.67. | 1. The CFA confirmed the preliminary validity of 12 of 20 factors initially found in the full EFA; 2. The CFA showed that the model including only the twelve confirmed factors (F2, F3, F4, F5, F7, F8–9, F10, F14, F16, F18, F19, F20) had excellent overall validity; 3. The total scale explained 50% of the variance and had a Cronbach alpha of 0.841 (95% CI: 0.819–0.859). All of the factors except F9-Fatalistic Attitude had a Cronbach alpha above 0.8. |
| Three surveys conducted with the national cancer screening programs [26-28] | China | Cancer Screening  Program in Rural Areas  Cancer Screening Program in Urban China  and Cancer Screening  Program in  Huaihe River  Areas | Chinese | Population at risk of cancer according to the protocols of the cancer screening programs  (1) general population who have never participated in any cancer screening programs at a community-level; (2) individuals who have previously attended the program for cancer risk assessment or screening intervention;  (3) cancer patients who were receiving treatment in local hospitals;  (4) a special group from employees of government and public institutions (non-health system), state-owned enterprises and private enterprises (to have better understand on the impact of socioeconomic factors)  1) permanent residency in the target counties or villages and aged 40 to 69 years, 2) no history of cancer, 3) signed informed consent form | 3,  6,  NA | Basic knowledge about cancer, primary prevention of cancer, secondary prevention of cancer  basic information, consciousness of common risk factors to cancer, awareness of early detection, awareness of early diagnosis, awareness of early treatment, and the needs and approaches for knowledge of cancer prevention and treatment  1) and 2) awareness of the disease burden of cancer incidence and cancer mortality, respectively; 3) 4 items on the 10 most common cancers in the Chinese population; 4) awareness of trends in cancer incidence since the 1970s for the Chinese population; 5) and 6) 4 items on awareness of risk factors and related preventive measures, respectively; 7) 5 items on when to seek medical advice (questions about each of the warning signs and 1 open-ended question); and 8) and 9) items related to awareness that cancer can be prevented and treated, respectively | 13  49  9 | None of these questionnaires was assessed. | For the questionnaire used in the Cancer Screening Program in Urban China, high cancer prevention knowledge scores were inversely associated with the overall risk of cancer. |
